# Clinical performance of blood biomarkers across settings and countries

**DOI:** 10.1002/alz70856_097509

**Published:** 2025-12-24

**Authors:** Sebastian Palmqvist, Noëlle Warmenhoven, Federica Anastasi, Andrea Pilotto, Shorena Janelidze, Pontus Tideman, Erik Stomrud, Niklas Mattsson‐Carlgren, Rik Ossenkoppele, Henrik Zetterberg, Joel B. Braunstein, Philip B. Verghese, Silke Kern, Kaj Blennow, Nicholas J. Ashton, Marc Suárez‐Calvet, Oskar Hansson

**Affiliations:** ^1^ Lund University, Lund, Sweden; ^2^ Barcelonaβeta Brain Research Center (BBRC), Pasqual Maragall Foundation, Barcelona, Spain; ^3^ University of Brescia, Brescia, Italy, Italy; ^4^ Department of Psychiatry and Neurochemistry, University of Gothenburg, Gothenburg, Sweden; ^5^ C2N Diagnostics, LLC, St. Louis, MO, USA; ^6^ C2N Diagnostics, LLC, Saint Louis, MO, USA; ^7^ University of Gothenburg, Gothenburg, Sweden; ^8^ University of Gothenburg, Mölndal, Sweden; ^9^ Banner Alzheimer's Institute and University of Arizona, Phoenix, AZ, USA; ^10^ Barcelonaβeta Brain Research Center (BBRC), Barcelona, Spain

## Abstract

**Background:**

Several blood‐based biomarkers for Alzheimer's disease (AD) are now becoming available for clinical practice. However, significant knowledge gaps remain that need to be addressed. In this study, we evaluated fully automated immunoassays and mass spectrometry‐based assays for phospho‐tau217 (*p*‐tau217), Aβ42, and Aβ40, specifically designed for use in clinical practice.

**Method:**

The study included 1,767 participants with cognitive symptoms from secondary care across four independent cohorts: Malmö, Sweden (*n* = 337), Gothenburg, Sweden (*n* = 165), Barcelona, Spain (*n* = 487), and Brescia, Italy (*n* = 230). Additionally, a primary care cohort from Sweden was included (*n* = 548). Plasma *p*‐tau217 and Aβ42 were measured using the Lumipulse immunoassays (Fujirebio). The *p*‐tau217 to non‐p‐tau217 (%p‐tau217) and Aβ42/40 ratios was measured using mass spectrometry‐based assays (PrecivityAD2; C2N Diagnostics). The primary outcome was AD pathology determined by the cerebrospinal fluid Aβ42/p‐tau181 ratio.

**Result:**

Plasma *p*‐tau217 (Lumipulse) detected AD pathology with AUCs of 0.93‐0.96. In secondary care, the accuracies were 89‐91%, the positive predictive values (PPV) 89‐95%, and the negative predictive values (NPV) 77‐90%. In primary care, the accuracy was 85%, the PPV was 82%, and the NPV was 88%. Accuracy was lower in participants over 80 (83%), but unaffected by chronic kidney disease, diabetes, sex, *APOE* genotype or cognitive stage. Using a two‐cutoff approach, accuracies increased to 92‐94% in secondary and primary care, excluding 12‐17% with intermediate results. Using the plasma *p*‐tau217/Aβ42 ratio did not improve accuracy but reduced intermediate test results (≤10%). %p‐tau217 (mass spectrometry) demonstrated similar accuracies in secondary care settings but showed higher accuracy in primary care. Notably, its performance was unaffected by age.

**Conclusion:**

Both the mass spectrometry‐based and fully automated assays demonstrated suitable performance for clinical implementation. However, a two‐cutoff approach may be required in specific settings and subpopulations, particularly for the fully automated assays. Findings on clinicians' attitudes, the impact of PrecivityAD2 on clinical care, and comparisons between blood and cerebrospinal fluid (CSF) biomarkers using amyloid PET as the reference standard will also be presented.

